# Reduced response to regadenoson with increased weight: An artificial intelligence–based quantitative myocardial perfusion study

**DOI:** 10.1016/j.jocmr.2024.101066

**Published:** 2024-07-25

**Authors:** Emmanouil Androulakis, Georgios Georgiopoulos, Alessia Azzu, Elena Surkova, Adam Bakula, Panagiotis Papagkikas, Alexandros Briasoulis, Ranil De Silva, Peter Kellman, Dudley Pennell, Francisco Alpendurada

**Affiliations:** a Royal Brompton and Harefield Hospitals, Guy’s & St Thomas’ NHS Foundation Trust, London, UK; b School of Biomedical Engineering and Imaging Sciences, Kings College London, London, UK; c Department of Clinical Therapeutics, School of Medicine, Alexandra General Hospital, National and Kapodistrian University of Athens, Athens, Greece; d National Heart and Lung Institute, Imperial College London, London, UK; e National Heart, Lung, and Blood Institute, National Institutes of Health, Bethesda, Maryland, USA

**Keywords:** Cardiovascular magnetic resonance, Regadenoson, Adenosine, Weight, Quantitative perfusion

## Abstract

**Background:**

There is conflicting evidence regarding the response to a fixed dose of regadenoson in patients with high body weight. The aim of this study was to evaluate the effectiveness of regadenoson in patients with varying body weights using novel quantitative cardiovascular magnetic resonance (CMR) perfusion parameters in addition to standard clinical markers.

**Methods:**

Consecutive patients with typical angina and/or risk factors for coronary artery disease (N = 217) underwent regadenoson stress CMR perfusion imaging using a dual-sequence quantitative protocol with perfusion parameters generated from an artificial intelligence (AI)–based algorithm. CMR was performed on 1.5T scanners using a standard 0.4 mg injection of regadenoson. A cohort of consecutive patients undergoing adenosine stress perfusion (N = 218) was used as a control group.

**Results:**

An inverse association of myocardial perfusion reserve and weight (mean decrease −0.05 per 10 kg increase, 95% confidence interval [CI] −0.009/−0.0001, P = 0.045) was noted in the regadenoson group but not in patients stressed with adenosine (P = 0.77). Adjusted logistic regression analysis revealed a 10 kg increase resulted in 36% increased odds for inadequate stress response (odds ratio [OR] = 1.36, 95% CI 1.10–1.69, P = 0.005). Moreover, a significant interaction (OR = 1.09, 95% CI 1.02–1.16, P = 0.012) between stressor type (regadenoson vs adenosine) and weight was noted. This was also confirmed in the propensity-matched subgroup (P = 0.024) and was not attenuated after adjustment (P = 0.041). Body surface area (BSA) (P = 0.006) but not body mass index (P = 0.055) was differentially associated with inadequate response conditional to the stressor used, and this association remained significant after adjustment for confounders (P = 0.025). Patients in the highest quartile of weight (>93 kg) or BSA (>2.06 m^2^) had substantially increased odds for inadequate response with regadenoson (OR = 8.19, 95% CI 2.04–32.97, P = 0.003 for increased weight and OR = 7.75, 95% CI 1.93–31.13, P = 0.004 for increased BSA). Both weight and BSA had excellent discriminative ability for inadequate regadenoson response (receiver operating characteristic area under curves 0.84 and 0.83, respectively).

**Conclusion:**

Using quantitative perfusion CMR in patients undergoing pharmacological stress with regadenoson, we found an inverse relationship between patient weight and both clinical response and myocardial perfusion parameters. A fixed-dose bolus approach may not be adequate to induce maximal hyperemia in patients with increased weight. Weight-adjusted stressors, such as adenosine, may be considered instead in patients with body weight >93 kg and BSA >2.06 m^2^.

## Graphical abstract

**Figure:**
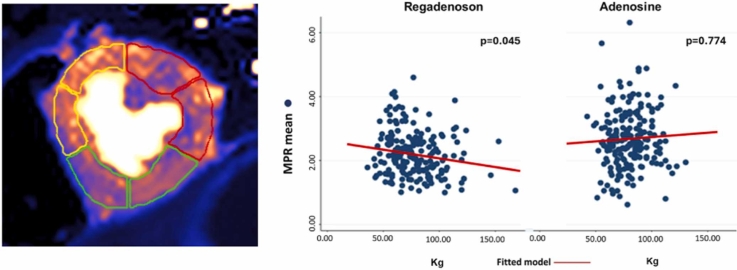
Using quantitative perfusion CMR (left panel, example of an AI segmented perfusion map), we found an inverse association of MPR and weight in 217 consecutive patients on fixed-dose regadenoson, but not with adenosine (right panel). Regadenoson may not be adequate to induce maximal hyperemia in patients with increased weight. Weight-adjusted stressors such as adenosine may be considered instead.

## Abbreviations

AI: artificial intelligence
AIF: arterial input function
AUC: area under the curve
BMI: body mass index
BP: blood pressure
BSA: body surface area
CAD: coronary artery disease
CMR: cardiovascular magnetic resonance
FOV: field of view
HASTE: half Fourier acquisition single-shot turbo spin-echo
HR: heart rate
IQR: interquartile range
LGE: late gadolinium enhancement
MBF: myocardial blood flow
MPR: myocardial perfusion reserve
ROC: receiver operating characteristic
SD: standard deviation
SPECT: single-photon emission computerized tomography
SSFP: steady state-free precession
TE: echo time
TI: inversion time
TR: repetition time
mcg: micrograms
CI: confidence interval
OR: odds ratio

## 1 Background

Myocardial perfusion cardiovascular magnetic resonance (CMR) is well established for the assessment of myocardial ischemia, having demonstrated high diagnostic accuracy in patients with coronary artery disease (CAD) [1], [2], [3], and additional predictive value for adverse cardiovascular outcomes [4], [5]. It relies on the evaluation of first-pass perfusion of gadolinium-based contrast agents under pharmacological vasodilation [6]. The most commonly used stressors are adenosine and regadenoson.

Adenosine has been the reference coronary vasodilator stress agent; the standard protocol mandates dose adjustment according to the patient’s weight and potential for further dose increase (from 140 up to 210 micrograms (mcg)/kg/min) according to the hemodynamic response to adenosine during infusion [7]. Due to non-selective activation of all adenosine receptors (A1, A_2A_, A_2B_, and A3), it has potential side effects and is contraindicated in patients with active or severe bronchoconstrictive disease. It requires a continuous 3–4 min intravenous infusion before first-pass perfusion images can be acquired and ideally the use of separate cannulae for the vasodilator and the gadolinium contrast agent.

Regadenoson, a selective A_2A_-receptor agonist, has emerged as a useful alternative when adenosine is contraindicated. It has a more favorable safety profile and is administered as a single intravenous bolus injection with only one cannula being used for both vasodilator and contrast agent, facilitating patient comfort and clinical workflow [8], [9]. A fixed standard dose of regadenoson is used (400 mcg), with the practical advantage of no dose calculation or adjustment according to the patient’s weight. The concept of a fixed dose has been initially supported by previous data [10]. However, this potentially raises concerns about suboptimal dosing in the increasingly overweight adult population. A recent study on the safety of regadenoson has documented blunted heart rate (HR) response in patients with high body mass index (BMI) (>30 kg/m^2^) [9]. Of note, a weight-based dose of regadenoson (8 mcg/kg) is currently being used in pediatric patients and has been demonstrated to be both feasible and well-tolerated for perfusion CMR [11].

The purpose of this study was to compare the effectiveness of regadenoson in patients with varying body weights using novel quantitative CMR perfusion parameters in addition to standard clinical markers using adenosine as a benchmark.

## 2 Methods

### 2.1 Study design

This was an observational single-center study of consecutive patients with angina and/or suspected or known CAD above 18 years old who were recruited at the Royal Brompton Hospital, London, United Kingdom, between January 2019 and July 2021 for CMR evaluation of myocardial perfusion.

### 2.2 Data collection

Two hundred and seventeen consecutive patients underwent stress CMR perfusion scans using regadenoson as a stressor at the standard dose of 400 mcg. Two hundred and eighteen consecutive patients underwent conventional, weight-adjusted, continuous infusion adenosine protocol as a control group. All examinations were performed at 1.5T (Magnetom Aera or Avanto, Siemens, Germany). Before examination, a 20-gauge intravenous catheter was placed in an antecubital vein for contrast agent injection (Gadobutrol, Bayer AG, Germany). In the adenosine group, hyperemia was induced using adenosine infusion via a peripheral cannula at a minimum rate of 140 mcg/kg/min for 4 min while patient’s symptoms, HR, and blood pressure (BP) were recorded. For perfusion quantification with adenosine, stress perfusion imaging was performed before rest perfusion imaging. Conversely, in the regadenoson group, resting perfusion imaging was performed before regadenoson stress imaging to avoid confounding effects from regadenoson infusion, as it has been previously showed that resting perfusion obtained 20 min after regadenoson with aminophylline reversal was higher than rest perfusion performed before regadenoson [12]. Clinical response was defined as an HR increase >10 bpm or a systolic BP drop >10 mmHg during pharmacological stress [7]. No significant adverse events were noted in either group.

### 2.3 Baseline CMR study and perfusion imaging

Multislice spin-echo images (half-Fourier acquisition single-shot turbo spin-echo) were obtained in three orthogonal planes to define the cardiac anatomy and to guide image acquisition. Imaging protocols included electrocardiogram-gated steady-state free precession (SSFP) breath-hold cines for assessment of biventricular volumes and function. Typical imaging parameters were 1.12 ms echo time (TE), 2.65 ms repetition time (TR), 192 × 192 matrix, spatial resolution 1.9 × 1.9 mm^2^; flip angle 80°, 360 × 360 mm^2^ field of view (FOV), 8 mm slice thickness with 2 mm gap.

Patients were asked to refrain from caffeine for at least 24 h before the scan. Basal, mid-ventricular, and apical short-axis perfusion images were acquired both at rest and during hyperemia using a dual-bolus protocol and a time interval of 10–15 min between acquisitions. Image acquisition was performed over 60 heartbeats with a bolus of 0.05 mmol/kg gadobutrol administered at 4 mL/s followed by a 20 mL saline flush during the acquisition of the perfusion sequence. The perfusion sequence was developed by Kellman et al. [13], consisting of a dual-sequence approach with a low-resolution arterial input function (AIF) acquisition followed by a high-resolution myocardial perfusion acquisition. For the AIF acquisition, only the mid slice was acquired every RR interval, with a saturation prepared, low-resolution image with fast low-angle shot readout, acquisition matrix 48 × 64, temporal resolution 60 ms. For the myocardial perfusion acquisition, three short-axis slices as described above were acquired every RR interval using an SSFP readout, matrix 192 × 111, typical FOV 360 × 270 × 8 mm^3^, resolution 1.9 × 2.4 mm^2^, TE 1.04 ms, TR 2.5 ms, flip angle 50°, and total duration 142 ms per slice.

Myocardial perfusion maps were automatically generated and segmented into a 16-segment model using artificial intelligence (AI), with each pixel representing average myocardial blood flow (MBF) in mL per gram per minute. The AI tool uses a convolution neural net approach to delineate the left ventricle cavity and myocardium, excluding myocardial fat and papillary muscles as previously described [4]. As the contours were performed by AI without the need for user input, the perfusion data were blinded to other CMR and demographic parameters. Global MBF in mL/g/min was calculated by averaging MBF across the three slices. Myocardial perfusion reserve (MPR) was also automatically calculated, defined as the ratio between global MBF at stress over rest.

Late gadolinium enhancement (LGE) was performed at least 5 min following the second gadolinium bolus injection for stress perfusion imaging, using phase-sensitive inversion recovery sequences. Images were acquired in standard long-axis planes and consecutive short-axis slices (8 mm slice thickness with 2 mm gap; FOV 360 × 360 mm^2^; spatial resolution 1.4 × 1.4 mm^2^; matrix 256 × 256; TE 1.19 ms; TR 904 ms) in two-phase encoding directions. Inversion times were optimized to null the myocardium (290–400 ms) [14].

### 2.4 Ethics approval

The study was registered as a Clinical Audit by the Quality and Safety Department of Royal Brompton Hospital and data collection was in accordance with local research governance policy. Data collection was also in compliance with the Data Protection Act ensuring patient anonymity and protection of personal details.

## 3 Statistical analysis

Baseline characteristics of patients with regadenoson vs adenosine were compared with Pearson chi-square, Student t, or Wilcoxon rank sum tests as appropriate. Continuous variables are presented as mean ± standard deviation (SD) or median and interquartile ranges. Comparison of two groups was performed by unpaired Student’s t-tests or Mann-Whitney U tests. Categorical variables were presented as percentages and were compared using χ^2^ test. Furthermore, we regressed inadequate response to stressor on obesity indices (weight, BMI, and body surface area [BSA]) by logistic regression analysis. Linear regression was used to evaluate the association of continuous responses with independent variables. To examine the interplay between obesity and the odds of inducing an inadequate stress response, we employed an interaction term between weight and adenosine/regadenoson.

Due to slight baseline differences between the groups (baseline HR and resting MBF) and to minimize selection bias, we implemented a probit regression model and calculated the propensity score for the conditional probability of stressor classification (adenosine vs regadenoson) in 218 and 217 patients undergoing stress CMR under adenosine infusion and intravenous bolus regadenoson, respectively. The model for calculating the propensity score for each subject included baseline HR and baseline mean blood flow. A 1:1 matching algorithm of the nearest neighbor with no replacement was used with a caliper of 0.2 * SD of the propensity score. We assessed covariate balance between matched patients by calculating i) Student’s t-tests for equality of means in the two samples and ii) the reduction in the standardized percentage bias (difference of the sample means in the matched and non-matched sub-samples as a percentage of the square root of the average of the sample variances).

Optimal cut-off values of weight and BSA considering inadequate stressor response were calculated by maximizing the product of the sensitivity and specificity using the Liu method in relevant receiver operating characteristic (ROC) curves. A sample size of 410 patients (our study included 435 participants) allocated in two groups (adenosine vs regadenoson) provided adequate power at 0.9 level to detect a minimum difference of 0.2 mL/g/min in MPR between groups. Measures of dispersion for MPR were derived from previously published data. The non-parametric Mann-Whitney test was used for pairwise comparison. Type I error was predefined at 0.05. We deemed statistical significance at P < 0.05. All tests were two-tailed. All statistical analyses were carried out with STATA 16 (version IC 16.1, StataCorp LLC).

## 4 Results

The study cohort comprised 435 patients with a mean age of 59.5 years and a mean weight of 79.5 kg. One hundred and fifteen patients (26%) had baseline CAD and 22% had prior intervention (Table 1). Hyperemic HR was significantly higher in the regadenoson group (96.5 vs 89.4 bpm, P < 0.001) as well as hyperemic HR increase (26.2 vs 23.1 bpm, P = 0.027) compared to the adenosine group (Table 2).

**Table 1:** Baseline characteristics between adenosine and regadenoson groups.

	Regadenoson N = 217	Adenosine N = 218	P-value
Age (years)	58.4 (16.7)	60.6 (15.0)	0.158
Weight, kg	79.3 (21.6)	79.9 (16.3)	0.762
BSA m^2^	1.89 (0.28)	1.92 (0.22)	0.193
Hypertension %	45	67	0.001
Diabetes mellitus %	23	30	0.137
Hyperlipidaemia %	43	57	0.024
Baseline CAD %	23	30	0.067
Previous intervention %	16	29	0.152
Stable symptoms %	65	59	0.204
LVEF%	63.8 (12.1)	61.2 (11.9)	0.079
RVEF%	61.4 (8.4)	60.4 (8.3)	0.235
Presence of LGE %	51	50	0.886

Continuous variables are reported as mean (SD). Categorical variables are reported as percentages.

*BSA* body surface area*, CAD* coronary artery disease*, LVEF* left ventricular ejection fraction*, RVEF* right ventricular ejection fraction*, LGE* late gadolinium enhancement.

**Table 2:** Hyperemia and stress quantification between adenosine and regadenoson groups.

	Regadenoson N = 217	Adenosine N = 218	P-value
Baseline systolic BP (mmHg)	134.5 (23.5)	132.7 (21.1)	0.447
Baseline diastolic BP (mmHg)	75.3 (13.0)	76.9 (12.8)	0.113
Hyperemic systolic BP (mmHg)	128.9 (22.0)	128.9 (24.4)	0.983
Hyperemic diastolic BP (mmHg)	70.4 (14.1)	73.3 (15.5)	0.051
Systolic BP fall (mmHg)	6.3 (16.7)	5.6 (18.1)	0.205
Diastolic BP fall (mmHg)	5.0 (11.9)	4.3 (14.9)	0.634
Baseline heart rate (bpm)	70.5 (13.3)	65.7 (12.9)	<0.001
Hyperemic heart rate (bpm)	96.5 (16.1)	89.4 (15.9)	<0.001
Heart rate increase (bpm)	26.2 (13.6)	23.1 (15.1)	0.027
Resting MBF (mL/g/min)	1.00 (0.31)	0.86 (0.32)	<0.001
Stress MBF (mL/g/min)	2.13 (0.74)	2.21 (0.78)	0.181
MPR	2.23 (0.69)	2.70 (0.91)	<0.001

Variables are reported as mean (SD).

*BP* blood pressure*, MBF* myocardial blood flow*, MPR* myocardial perfusion reserve*.*

### 4.1 Effect of weight on stress response

**Fig. 1:**
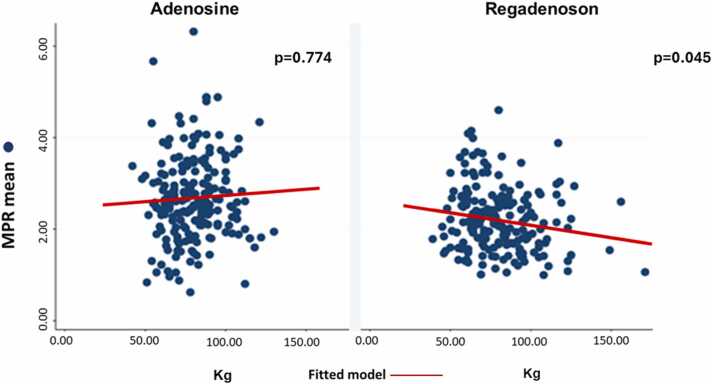
Correlation between weight and MPR in the regadenoson and adenosine groups. *MPR* myocardial perfusion reserve.

**Fig. 2:**
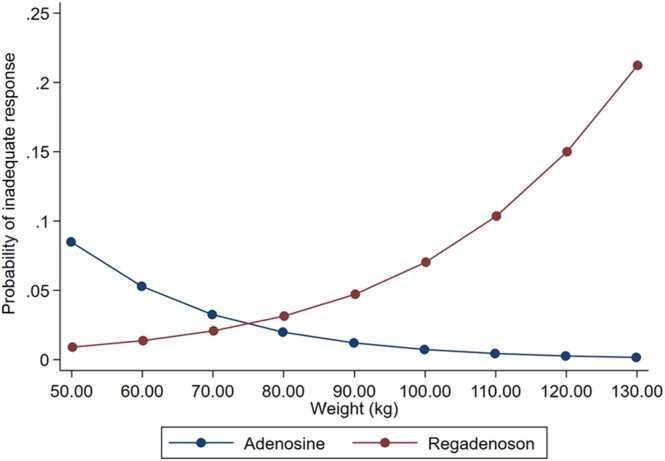
Differential association of weight with the probability of inadequate stress response conditional on adenosine vs regadenoson use.

**Fig. 3:**
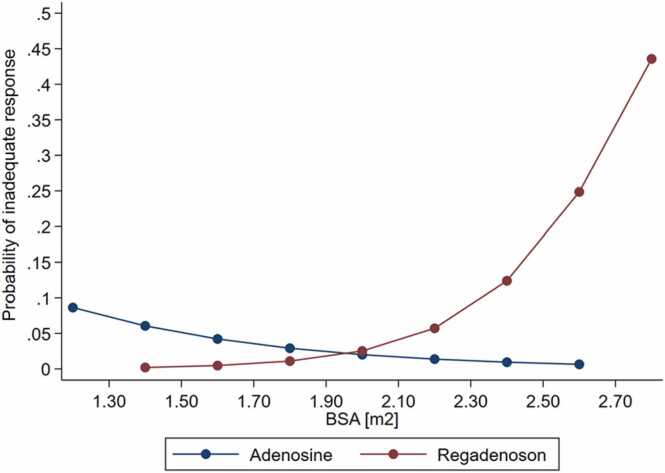
Differential association of BSA with the probability of inadequate stress response conditional on adenosine vs regadenoson use. *BSA* body surface area.

By linear regression analysis, there was an inverse association of MPR and weight (mean decrease −0.05 per 10 kg increase, 95% confidence interval [CI] −0.00993 to −0.00012, P = 0.045) in the regadenoson group but not in patients stressed with adenosine (95% CI −0.00736 to 0.00988, P = 0.774, Fig. 1). Increased weight was also associated with higher likelihood of inadequate clinical stress response in the regadenoson group (P = 0.001) but not in the adenosine group (P = 0.215). By logistic regression analysis, each 10 kg increase resulted in 36% increased odds for inadequate response (odds ratio [OR] = 1.36, 95% CI 1.10–1.69, P = 0.005) in patients stress with regadenoson. This result remained significant after controlling for age, gender, presence of infarction, hypertension, and hyperlipidemia (P = 0.022). We solidified the selective association of increased weight with higher odds of inadequate response in the regadenoson group only (P = 0.003) through a propensity-matched analysis (n = 308, 154 patients stressed with adenosine and 154 counterparts with regadenoson) for resting HR and MBF (Supplementary Table). The differential association of weight with inadequate stress response depending on the stressor was further evidenced by a significant interaction (OR = 1.09, 95% CI 1.02–1.16, P = 0.012) between stressor type (regadenoson vs adenosine) and weight. This interaction was also confirmed in the propensity-matched subgroup (P = 0.024) and was not attenuated after adjustment for age, gender, hypertension, hyperlipidemia, and previous infarction (P = 0.041). In other words, patients stressed with regadenoson had increased odds of experiencing reduced response as their weight increased compared to their odds of inadequate response under adenosine (ratio of OR, Fig. 2). Similarly, BSA was differentially associated with inadequate response conditional to the stressor used (P = 0.006); this association did not materially change after controlling for age, gender, hypertension, hyperlipidemia, and previous infarction (P = 0.025, Fig. 3). Moreover, BMI was also differentially associated with inadequate response conditional to stressor used in the propensity-matched subgroup (P = 0.055); this association however was attenuated after controlling for age, gender, hypertension, hyperlipidemia, and previous infarction (P = 0.128).

### 4.2 Relevant cut-offs

**Fig. 4:**
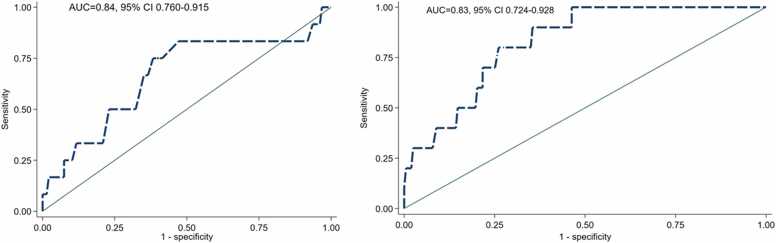
Receiver operating curves of weight (left panel) and BSA (right panel) for inadequate clinical response. *BSA* body surface area, *AUC* area under the curve, *CI* confidence interval.

Given the increased likelihood of inadequate response in patients stressed with regadenoson, we sought to derive clinically relevant cut-offs for weight and BSA. Notably, patients in the highest quartile of weight (>93 kg) or BSA (>2.06 m^2^) had substantially increased odds for inadequate stressor response with regadenoson (OR = 8.19, 95% CI 2.04–32.97, P = 0.003 for increased weight and OR = 7.75, 95% CI 1.93–31.13, P = 0.004 for increased BSA, respectively). ROC analysis showed similar evidence; both weight and BSA had excellent discrimination ability (area under curve 0.84 and 0.83, respectively) for inadequate response in patients stressed with regadenoson (Fig. 4).

## 5 Discussion

To the best of our knowledge, this is the largest study to date to show a reduced response to regadenoson with increased body weight using fully quantitative perfusion CMR indices. The higher the body weight, the lower the likelihood of response to regadenoson. This was supported by both hemodynamic and perfusion parameters.

### 5.1 Initial validation of regadenoson stress perfusion with nuclear imaging

As a selective A_2A_-receptor agonist, regadenoson has the potential to elicit a higher coronary hyperemic response with fewer side effects. ADVANCE MPI was a large multicenter double-blinded phase 3 trial evaluating patients who underwent sequential SPECT studies on different days with adenosine and regadenoson. This study showed that regadenoson was comparable to adenosine in detecting inducible ischemia by nuclear perfusion imaging [15]. Hemodynamic response, image quality, and perfusion scores were similar. Patients had fewer symptoms with regadenoson which was preferred as a stressor over adenosine. Higher tolerance in addition to a more convenient administration in the form of a single dose made regadenoson an attractive pharmacological stress agent to be used in myocardial perfusion studies.

The same investigators subsequently sought to determine if specific subsets of patients, such as those with obesity, had similar agreement with adenosine [10]. Patients were divided into those with BMI ≤30 (n = 770) and those with BMI >30 (n = 470). In this analysis, no significant difference in diagnostic agreement was found compared to adenosine, suggesting that regadenoson is as efficacious as adenosine in detecting ischemia regardless of BMI. Interestingly, the incidence of induced symptoms was similar in the group of patients with a BMI ≤30 kg/m^2^, but significantly lower with regadenoson in the group of patients with a BMI >30 kg/m^2^. Finally, it should be noted that BMI was chosen as a variable rather than weight. In our cohort, BMI was marginally significant in univariate models, but this was attenuated in multivariate models.

A limitation of the ADVANCE MPI trials is that two important patient subsets were excluded: i) patients who had contraindications to adenosine and ii) patients who had significant symptoms on the initial adenosine scan [10]. Overall, the ADVANCE trials showed regadenoson to be noninferior to adenosine for detecting myocardial ischemia. The overall visual agreement was comparably low (in the low 60% range) for the adenosine-regadenoson and adenosine-adenosine comparisons, both in qualitative studies and smaller quantitative study, using perfusion defect size (as a percentage) for quantification [16], [17].

### 5.2 CMR quantitative perfusion

Myocardial perfusion imaging using CMR has become an established non-invasive test for the detection of myocardial ischemia. Standard practice involves visual interpretation of a series of dynamic images and relies on experienced observers to identify inducible perfusion defects and hence myocardial ischemia. However, there is emerging interest in quantitative myocardial perfusion. Potential advantages over visual assessment include reduced operator dependence, simpler and faster analysis, and the ability to detect global disease rather than regional reduction of MBF [18]. The quantitative perfusion sequence used in this study has shown to be accurate for detecting hyperemia against coronary physiology assessment [19] and has shown to be an independent predictor of adverse cardiovascular outcomes [4]. Quantitative CMR perfusion therefore provides additional information to conventional visual assessment and is expected to play an important complementary role in the evaluation of myocardial ischemia.

### 5.3 Comparison with previous CMR studies

In a large study of consecutive patients undergoing regadenoson perfusion CMR, Monmeneu Menadas et al. documented a reduced HR response in patients with BMI ≥30 kg/m^2^ and diabetes [8]. However, this was a study focused on safety and tolerability to regadenoson only, and no data were available to further assess the effects of weight on myocardial perfusion. A different study looked specifically at the effects of obesity on quantitative CMR parameters [20]. Adenosine perfusion and regadenoson perfusion were performed sequentially in 28 healthy individuals. These subjects were divided into nonobese (BMI ≤30 kg/m^2^) and obese (BMI >30 kg/m^2^) groups. No significant difference in HR response was observed between groups. There was a mild negative association between MPR and BMI as assessed with both vasodilators (r = −0.37 with adenosine and r = −0.4 with regadenoson) which did not reach statistical significance. However, the study population was relatively small (12 obese patients out of a final cohort of 28), which may explain some of the differences compared with the current study.

## 6 Study limitations

This study was not randomized, which may explain the slight differences in baseline characteristics between the two groups as regadenoson was primarily used when adenosine was not feasible due to poor venous access or contraindicated. Moreover, the protocol used in this study was rest-stress imaging for regadenoson (no aminophylline was used to revert the effects of regadenoson) and stress-rest imaging for the adenosine control group. This protocol may be different from other institutions as there is no standard practice but may explain the small difference between myocardial rest blood flow between adenosine and regadenoson. However, these factors are unlikely to influence the key findings as enrollment was consecutive for each group. Secondary propensity-matched cohort analysis, used to minimize the selection bias, produced similar results thus supporting the original analysis (Supplementary Figure).

## 7 Conclusion

In this cohort of patients undergoing quantitative myocardial perfusion, we found that fixed-dose regadenoson was associated with reduced hemodynamic and hyperemic response with increasing body weight, while no such association was observed with weight-adjusted adenosine infusion. Regadenoson should be used with caution in patients with high body weight as the presence and severity of both territorial and microvascular ischemia may be underestimated in patients with increased body weight. A weight-adjusted stressor, such as adenosine, may be considered instead in patients with body weight >93 kg and BSA >2.06 m^2^.

## Funding

No funding.

## Author contributions

E.A. and F.A. conceived the study. E.A., G.G., A.A., E.S., A.B., and P.P. collected the data. E.A. and G.G. performed the statistical analysis. E.A., F.A., R.D.S., P.K., and D.P. interpreted the data. All authors contributed to the drafting and revising of the manuscript.

## Ethics approval and consent

The study was registered as a Clinical Audit by the Quality and Safety Department of Royal Brompton Hospital and data collection was in accordance with local research governance policy. Data collection was also in compliance with the Data Protection Act ensuring patient anonymity and protection of personal details.

## Consent for publication

Not applicable.

## Declaration of competing interests

The authors declare that they have no known competing financial interests or personal relationships that could have appeared to influence the work reported in this paper.

## Appendix A Supplementary material

.

**Supplementary Figure.** Differential association of weight with the probability of inadequate stress response conditional on adenosine vs regadenoson use in 308 matched patients.
