# Supplementary material for: Reduced response to regadenoson with increased weight: An artificial intelligence–based quantitative myocardial perfusion study
Source: J Cardiovasc Magn Reson. 2024 Jul 25;26(2):101066. doi: 10.1016/j.jocmr.2024.101066 (PMC11490868; doi:10.1016/j.jocmr.2024.101066)
Supplement: Supplementary file 1 — Supplementary material. [file mmc1.docx]

**Supplementary table**. Baseline characteristics and quantification parameters after propensity match.

| **Variable** | **Adenosine** | **Regadenoson** | **P-Value** |
| --- | --- | --- | --- |
| Age | 61.4 (14.1) | 59.5 (15.4) | 0.247 |
| Weight | 81.7 (16.9) | 83.5 (23.9) | 0.327 |
| BMI | 27 (4.65) | 28.2 (6.65) | 0.056 |
| BSA | 1.92 (1.72-2.06) | 1.92 (1.73-2.1) | 0.537 |
| LVEF | 65 (58-70) | 65.5 (57-71) | 0.297 |
| RVEF | 62 (57-67) | 61 (55-66) | 0.325 |
| Resting MBF | 0.86 (0.7-1.11) | 0.87 (0.73-1.01) | 0.989 |
| Stress MBF | 2.25 (1.63-2.78) | 1.97 (1.53-2.46) | 0.005 |
| MPR mean | 2.51 (2.02) | 2.24 (1.78) | 0.003 |
| Systolic BP stress | 128 (113-147) | 127 (115-142) | 0.444 |
| HR rest | 66.5 (59-75) | 64 (58-75) | 0.307 |
| HR stress | 90 (80-101) | 93 (83-103) | 0.173 |
| Systolic BP rest | 134 (119-154) | 132 (115-150) | 0.182 |
| Diastolic BP rest | 78 (70.5-86) | 74 (67-83) | 0.021 |
| HR increase | 22 (13-32) | 25.5 (19-34) | 0.005 |
| Variables are reported as mean (SD). BMI: body mass index; BSA: body mass index; LVEF: left ventricular ejection fraction; RVEF; right ventricular ejection fraction; MBF: myocardial blood flow; MPR: myocardial perfusion reserve; BP: blood pressure, HR: heart rate. | | | |
