# Supplementary figures and images for: Reduced response to regadenoson with increased weight: An artificial intelligence–based quantitative myocardial perfusion study
Source: J Cardiovasc Magn Reson. 2024 Jul 25;26(2):101066. doi: 10.1016/j.jocmr.2024.101066 (PMC11490868; doi:10.1016/j.jocmr.2024.101066)

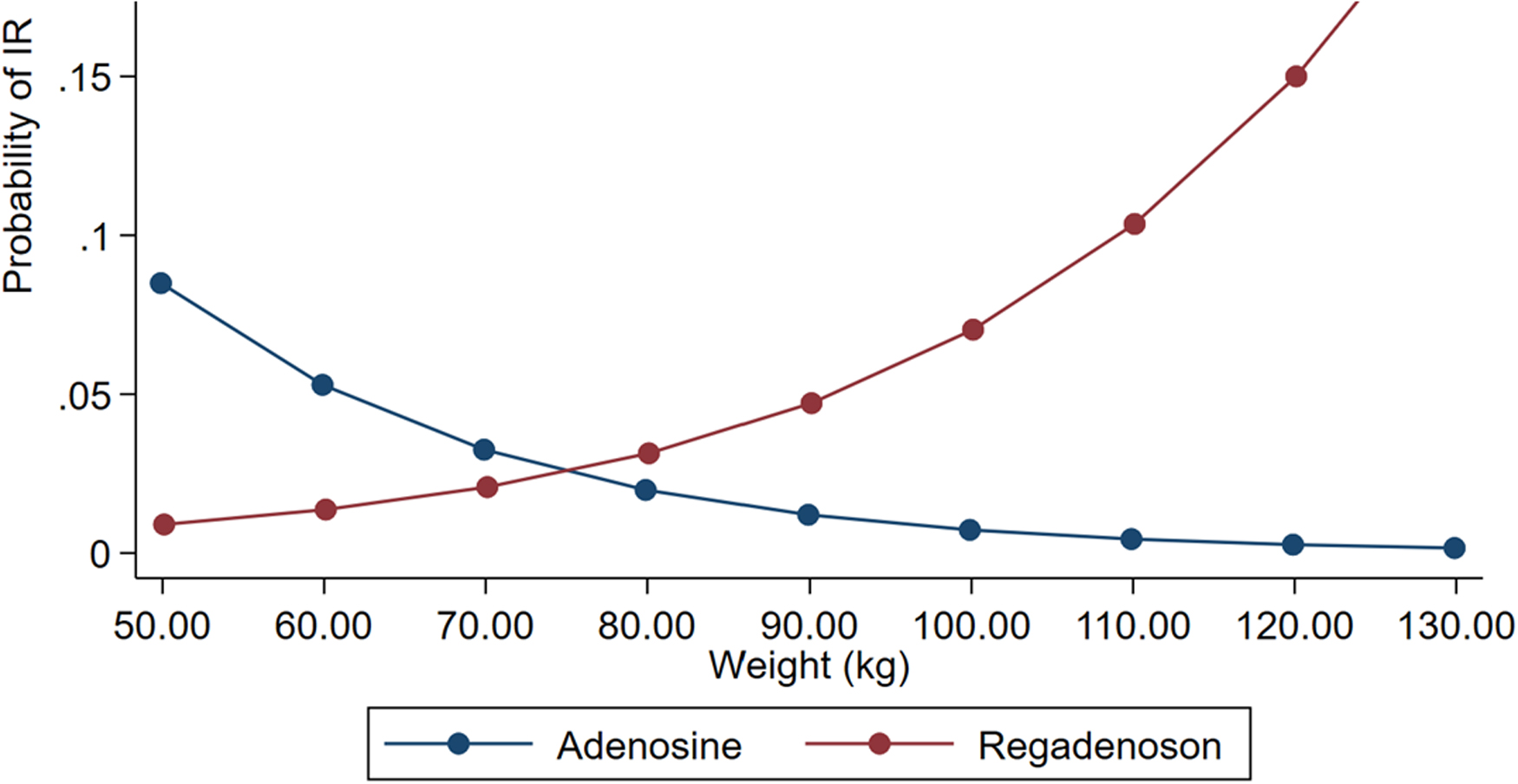

Supplement: Supplementary file 2 — Supplementary Figure. Differential association of weight with the probability of inadequate stress response conditional on adenosine vs regadenoson use in 308 matched patients. [file mmc2.jpg]
